# Hepatic Fat Quantification Using Beta Distribution and a Probabilistic Neural Network in a Prepubertal Male Cohort

**DOI:** 10.3390/diagnostics16101489

**Published:** 2026-05-14

**Authors:** Mario Alexis Ramírez-Bautista, Benito de Celis Alonso, Gerardo Uriel Pérez Rojas, Fernando Cocoletzi-Adame, Silvia S. Hidalgo Tobón, Moisés Arredondo-Velázquez, Eduardo Moreno-Barbosa, Javier M. Hernández-López, Po-Wah So, Jorge Velázquez-Castro

**Affiliations:** 1 Faculty of Physical and Mathematical Sciences, Meritorious Autonomous University of Puebla, Puebla 72570, Mexico; mario.ramirezba@alumno.buap.mx (M.A.R.-B.); bdca@fcfm.buap.mx (B.d.C.A.); emoreno@fcfm.buap.mx (E.M.-B.); javierh@fcfm.buap.mx (J.M.H.-L.); 2 Aicraft, Puebla 72590, Mexico; cocoletzi@aicraft.mx; 3 Imaging Department, Children Hospital of Mexico “Federico Gómez”, Mexico City 06720, Mexico; shid@xanum.uam.mx; 4 Physics Department, Metropolitan Autonomous University, Iztapalapa Unit, Mexico City 09310, Mexico; 5 Center for Research and Advanced Studies of the National Polytechnic Institute (CINVESTAV), Ciudad Victoria 87130, Mexico; juan.arredondo@cinvestav.mx; 6 Department of Neuroimaging, Institute of Psychiatry, Psychology and Neuroscience, King’s College London, London SE5 9NU, UK

**Keywords:** liver fat, prepubertal, probabilistic neural network, convolutional neural network, beta distribution, confidence intervals

## Abstract

**Background/Objectives:** High liver fat content is closely associated with hepatic disease and multiple comorbidities including cancer, diabetes and cardiovascular accidents. Therefore, accurate quantification of hepatic steatosis, especially of borderline cases, is essential for clinical management. Although MRI non-invasively assesses hepatic steatosis, current approaches remain limited by the data variability introduced through use of region-of-interest measurements or classification models that predict discrete fat grades without providing uncertainty estimates. This study proposes a probabilistic approach for hepatic steatosis quantification based on combining a neural network and beta distribution, enabling prediction of hepatic fat percentage with corresponding confidence intervals. **Methods:** Single in-phase Dixon MRI liver images from a cohort of prepubertal males (n = 84) were used as input to a probabilistic neural network combined with a beta distribution framework to estimate hepatic fat content along with associated confidence intervals. The predicted fat fractions were then compared against reference MRI-derived measurements (ground truth). **Results:** The methodology achieved a low prediction error and demonstrated good performance for the test set, with predicted values in good agreement with the ground truth measurements. This was reflected by the mean absolute error (MAE = 0.44 percentage points) and the coefficient of determination (R^2^ = 0.98). The empirical standard deviation of the prediction errors on a logarithmic scale was σ = 0.0609. **Conclusions:** By incorporating uncertainty quantification into hepatic steatosis estimation, this probabilistic framework provides an interpretable measure of variability alongside point estimates. The approach is demonstrated in a specific cohort and requires further validation in broader populations.

## 1. Introduction

Metabolic Dysfunction-Associated Steatotic Liver Disease (MASLD) is the most common chronic liver disease worldwide, with an estimated global prevalence of around 25% [1,2]. MASLD initially involves abnormal hepatic fat accumulation (steatosis). Accurate quantification of hepatic steatosis is required for early diagnosis and clinical decision making, as accumulating hepatic fat is associated with disease progression towards fibrosis, cirrhosis and hepatocellular carcinoma [3,4]. Currently, liver biopsy is the gold standard for steatosis quantification [5]. However, its invasiveness limits its routine use, especially in pediatric and prepubertal populations [6]. Furthermore, biopsy only collects a small and localized portion of the liver, approximately 1/50,000 of the whole liver mass, while steatosis is variable over the whole organ, and biopsy inevitably leads to sampling bias. Thus, the development of non-invasive methods for diagnosis and quantification of liver fat is a necessity [7]. Magnetic resonance imaging (MRI) is non-invasive and does not use ionizing radiation, although is contraindicated for those with a pacemaker or metallic implants or who suffer from claustrophobia. Recent studies have shown that MRI measurements of hepatic fat correlate well with the degree of steatosis assessed by biopsy and MRI is increasingly considered a more appropriate modality for detecting excess hepatic fat [8,9,10,11,12,13,14].

Current clinical practice for MRI-based hepatic fat quantification often involves placing regions of interest (ROIs) on Proton Density Fat Fraction (PDFF) maps of the liver, carefully avoiding structures such as blood vessels, the gastrointestinal tract or bile ducts. However, the selection of a limited number of ROIs can introduce sampling bias, particularly given the heterogeneous distribution of hepatic fat in MASLD [8,15,16]. Up to 60% of patients with MASLD present a heterogeneous pattern of steatosis [17] and ROI selection may not be representative of the disease as a whole. Although standardized approaches have been proposed to reduce this variability, such as placing ROIs across the nine Couinaud liver segments [9], the manual placement and analysis of multiple ROIs is too time-consuming for routine clinical practice [17]. To overcome sampling limitations within MRI-based assessment, advanced techniques exploiting the chemical shift difference between water and fat, such as multi-echo Dixon methods, have been developed, enabling voxel-wise quantification of liver PDFFs across the entire liver [18]. These approaches correct for several confounding factors, including the spectral complexity of fat, and are therefore considered the MRI reference standard for liver fat quantification [18].

In recent years, deep neural networks (deep learning) have established their utility as powerful tools for the automated analysis of medical images [19]. However, most existing approaches, including classical methods that use average ROI measurements [9,17,20,21] as well as deep learning models, consider the problem either as a standard regression task that predicts a single deterministic hepatic fat percentage, or as a classification task that distinguishes between different steatosis grades [22,23,24,25]. These approaches overlook the fact that hepatic steatosis is inherently an estimate that should be accompanied by confidence intervals. By reporting only a single value, these methods fail to capture the variability in the measurement, which is crucial for clinical interpretation.

Probabilistic neural networks (PNNs) have been proposed as an alternative framework to enhance diagnostic accuracy [26]. Unlike conventional neural networks that aim to produce a single point estimate, PNN models learn the parameters of the target distribution, providing a probabilistic characterization of the outcome [27]. This formulation offers an important advantage for hepatic fat content; rather than directly inferring a single value, it estimates the underlying distribution from which the target values arise [26,28]. Once this distribution is learned, confidence intervals, uncertainty bounds, and other relevant statistics can be derived for each prediction, enabling a more informative interpretation of the model output.

For variables representing proportions bounded in the range (0, 1), such as PDFF, the beta distribution provides a natural probabilistic model. The beta distribution is a family of continuous probability distributions defined on the interval (0, 1) and parameterized by two positive shape parameters, α and β, which determine its form [29,30]. When combined with a PNN, estimating these parameters enables the model to capture both the most likely PDFF value and its associated confidence interval. Within this probabilistic framework, dispersion of the measurements can be evaluated using intervals based on the standard deviation of the underlying uncertainty distribution. Under well-calibrated conditions, approximately 68% and 95% of observations are expected to fall within ±1σ and ±2σ intervals respectively [31]. These intervals provide a simple and interpretable means of assessing the agreement between predicted values and ground truth measurements.

More broadly, probabilistic formulations of this type, often implemented Bayesian principles or techniques such as Bayesian optimization, have been successfully applied to the analysis of data from medical imaging modalities, including ultrasound, Computed Tomography (CT), and resting state functional magnetic resonance imaging (rs-fMRI) [28,32,33]. These approaches have been particularly valuable in data-limited settings, where their ability to model uncertainty can help mitigate overfitting and improve the robustness of predictions [34]. Nevertheless, to the best of our knowledge, such approaches have not yet been applied to MRI-based estimation of hepatic fat.

This study proposes a PNN framework for hepatic fat quantification, motivated by recent evidence showing that combining convolutional neural network (CNN)-based feature extraction with probabilistic modeling enhances medical image analysis by leveraging hierarchical spatial representations and uncertainty-aware predictions [33]. The proposed method also provides confidence intervals and a full probability distribution, thereby capturing the uncertainty inherent for each estimate. In addition, PNN-based approaches are well suited to data-limited settings, as they incorporate probabilistic representations that help stabilize learning under data-constrained conditions.

## 2. Materials and Methods

### 2.1. Database

The volunteer dataset was retrospectively obtained from the METCOG project, funded by the Medical Research Council United Kingdom—CONACYT Research Partnerships Newton Fund 2015 Call. The cohort included 84 Latin American male prepubertal participants (aged 7 to 9 years) recruited from elementary schools in Mexico City who had undergone a liver MRI examination (see below) at the Infant Hospital of Mexico “Federico Gómez” (MIHFG). All participants were assessed using Tanner staging to confirm prepubertal status. Depression screening tests verified the absence of depressive symptoms.

### 2.2. MRI

MRI examinations were performed on a 3T Skyra scanner (Siemens^®^, Erlangen, Germany) using a whole-body coil. Briefly, liver fat quantification was performed using multi-echo Dixon acquisitions (Perspectum, LiverMultiScan^®^, Oxford, UK) [11,12,16] that exploit the chemical shift difference between water and fat to generate maps of the PDFF. Multiple echoes were acquired to obtain in-phase and out-of-phase signals, enabling the separation of water and fat components. Values of PDFF obtained from Liver Multi Scan (LMS) were used as ground truth in this study. Detailed MRI acquisition and processing parameters are described elsewhere [11].

### 2.3. Data Preparation

**Figure 1:**
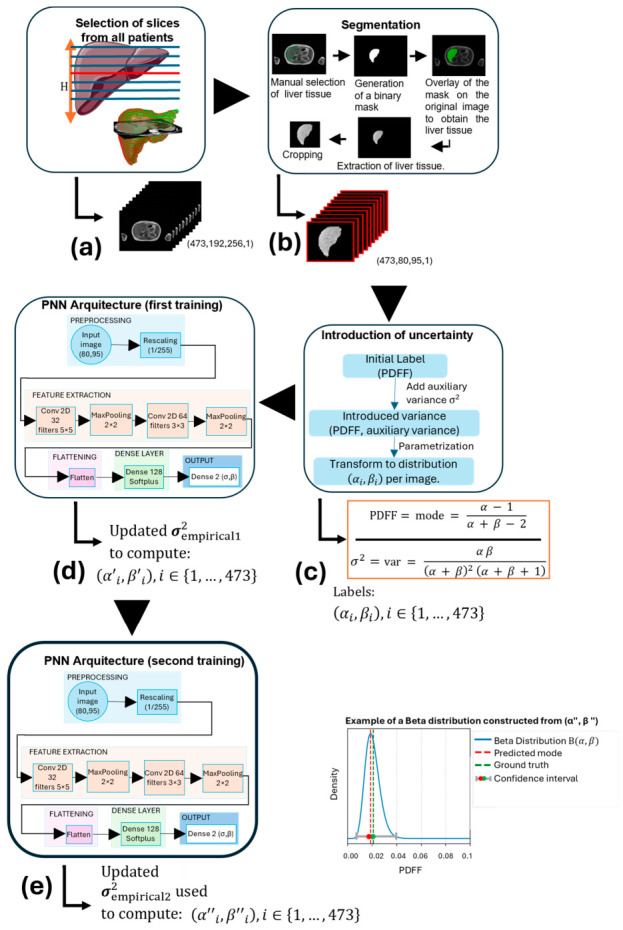
Overview of the proposed methodology. (**a**) Slice selection: A total of 473 images (192 × 256 pixels) were obtained from all patients. (**b**) Liver segmentation and preprocessing, yielding resized images (80 × 95 pixels). (**c**) Initial uncertainty modeling: PDFF labels were converted into beta parameters (*α_i_*, *β_i_*) using an auxiliary variance. (**d**) First training stage: The PNN was trained, an empirical variance (σ′^2^_empirical1_) was estimated from predicted modes and updated labels (*α*′, *β*′) were computed. (**e**) Second training stage: The network was retrained with updated labels, a second empirical variance (σ′^2^_empirical2_) was estimated, and final parameters (*α_i_*″, *β_i_*″) were obtained to derive PDFF confidence intervals.

Only the Dixon in-phase MRI data were inputted into the PNN. Given the spatial heterogeneity of hepatic fat distribution, relying on a single slice per subject may overlook local variations. Initial training experiments revealed data insufficiency, manifested as premature overfitting, motivating the adoption of a multi-slice strategy to improve robustness. Thus, a central slice containing liver parenchyma was selected, and additional 3–6 adjacent slices were included both superiorly and inferiorly, yielding 5–8 images per subject (Figure 1a). This approach provided a more representative sampling of the liver and supported more stable model training.

A total of 473 images with a resolution of 256 × 192 pixels were used from the 84 study participants. Manual segmentation was then carried out using Python’s OpenCV (v4.10.0) library to exclude non-liver structures (e.g., arms, abdominal fat, gallbladder, lungs, and intestines) and was performed by expert radiologists (Figure 1b). This segmentation procedure involved manual delineation of liver tissue, generation of a binary mask, overlay of the mask onto the original image to isolate the liver region, extraction of the segmented liver tissue, and final image cropping.

After segmentation, images were processed using the Pillow library (version 11.1.0) and resized to a uniform resolution of 80 × 95 pixels to standardize inputs for the machine learning models. The resulting dataset was then divided into training, validation, and test sets, comprising 331 images (70%), with 71 (15%) and 71 (15%), respectively, for model development and evaluation.

### 2.4. Modeling of PDFF

The PNN was designed to estimate the parameters of a beta distribution describing liver fat content. From the predicted distribution parameters, both a point estimate of the PDFF, corresponding to the mode of the distribution, and confidence intervals describing measurement uncertainty can be derived [35,36].

PDFF values provided by LMS analysis were assumed to correspond to the mode of an underlying beta distribution. However, since this scalar measurement does not uniquely determine the (*α*, *β*) parameters (see Supplementary Information 1), an auxiliary variance of 0.0025 was introduced to construct the initial training labels (*α_i_*, *β_i_*). This variance represents an assumed level of measurement uncertainty and enables the definition of a plausible beta distribution around each reported PDFF value. In practice, this parameter acts as an implicit regularizer, introducing a hyperparameter that controls the dispersion of the target distribution, helps prevent ill-posed solutions, and improves training stability by attenuating overfitting via avoiding overly concentrated target distributions (Figure 1c). It should be noted that in this step, this variance was not fully learned from the data. These labels were used for the initial training phase, after which an empirical variance (*σ*′^2^_empirical1_) was estimated from the dispersion between predicted values and ground truth (LMS) values in the training set. This subsequent estimate allowed the model to partially adapt the dispersion based on observed data (Figure 1d). To ensure that this uncertainty reflected, to a first approximation, the intrinsic variability in the measurement process rather than being solely attributed to model error, variance estimated from model residuals was incorporated during training as a calibration factor, with model calibration subsequently assessed on the test set. This empirical derived variance was assigned uniformly across all samples, allowing each observation to retain a distinct mode while sharing a common variance. The updated variance was then used to recompute the distribution parameters, yielding revised labels (*α_i_*′, *β_i_*′) for each image. Subsequently, these revised labels (*α*′, *β*′) were used to retrain the network (Figure 1e). After retraining, a second empirical variance (*σ*′^2^_empirical2_) was estimated using the same procedure. This variance was then again used to recompute the corresponding (*α_i_*″, *β_i_*″) parameters. These final parameters were then used to derive PDFF confidence intervals from the beta distribution and to quantify predictive uncertainty in the test set.

To reduce the risk of overfitting given the limited number of subjects, the regularization induced by the auxiliary variance was combined with patient-level data splitting and the use of a PNN, as described in the following section.

### 2.5. Neural Network Architecture

The input to the PNN consisted of normalized images of size (80 × 95 × 1), scaled to the range [0, 1] to ensure numerical stability (Figure 1d,e). The network architecture was implemented as a lightweight CNN, designed to learn the mapping to the corresponding (*α*, *β*) parameters from input images, while mitigating overfitting given the limited dataset size. The resulting representations were extracted through two convolutional blocks. The first block employed 32 filters of size 5 × 5 to capture coarse spatial patterns, followed by max pooling for dimensionality reduction. The second block with 64 filters of size 3 × 3 was then used to extract finer hierarchical features from hepatic tissue. This progressive increase in filter depth followed standard CNN design principles, allowing the network to learn increasingly abstract representations. The extracted features were flattened and mapped to a latent space through a fully connected layer with 128 neurons and softplus activation [37], providing a compact and smooth representation. The model subsequently predicted the (*α*, *β*) parameters of a beta distribution. To ensure valid and unimodal distributions, a softplus transformation followed by an additive shift was applied, enforcing *α*, *β* > 1. Optimization was performed using the Adam optimizer to minimize the Mean Squared Error (MSE) loss, with a learning rate of 1 × 10^−3^, a batch size of 16 and 25 training epochs.

## 3. Results

### 3.1. Model Training and Performance Evaluation

**Figure 2:**
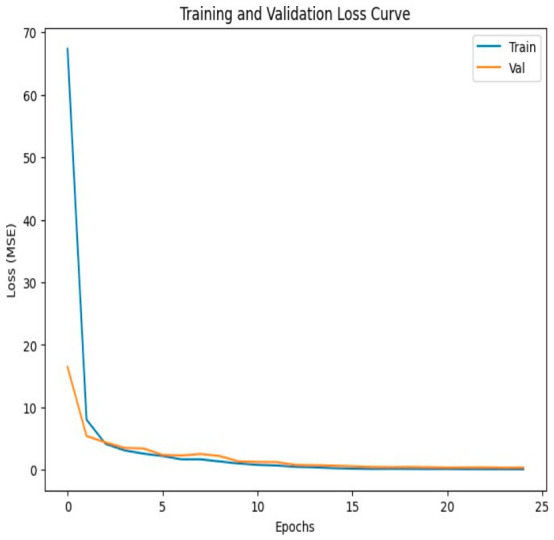
Loss (MSE) over 25 training epochs. Results are presented for the training and validation test groups. These calculations correspond to the second training stage (Figure 1e).

Model performance during training was evaluated by monitoring the MSE loss for both the training and validation datasets across 25 epochs. As learning progressed, a decrease in the loss values was expected. The curves corresponding to the second training stage are shown in Figure 2.

**Figure 3:**
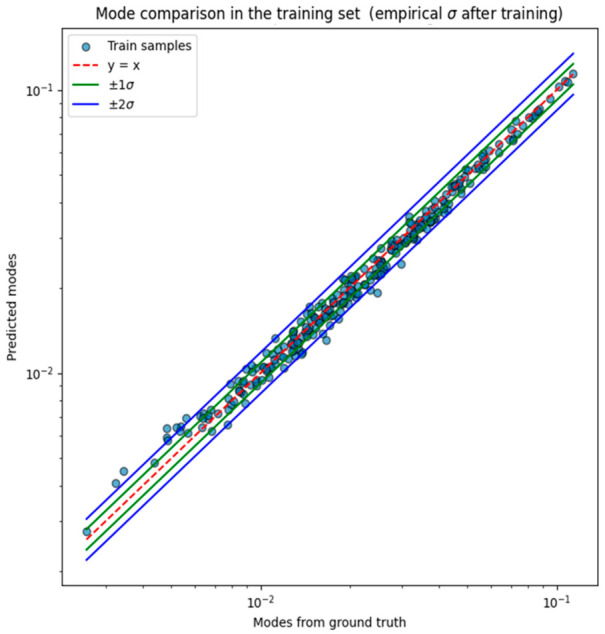
Model-predicted modes vs. ground truth modes derived from LMS PDFF measurements. Data presented for the training set after the second training stage.

The correlation between the predicted modes (corresponding to PDFF) and the modes derived from the ground truth values for the training set are presented in Figure 3. The empirical standard deviation of the prediction errors, computed in logarithmic scale, was σ = 0.0609 and was used to construct ±1σ and ±2σ intervals. The logarithmic scale was adopted because most predicted and ground truth PDFF modes were concentrated in the low value range and clustered near zero.

### 3.2. Quantitative Evaluation on the Test Set and Uncertainty Quantification

**Figure 4:**
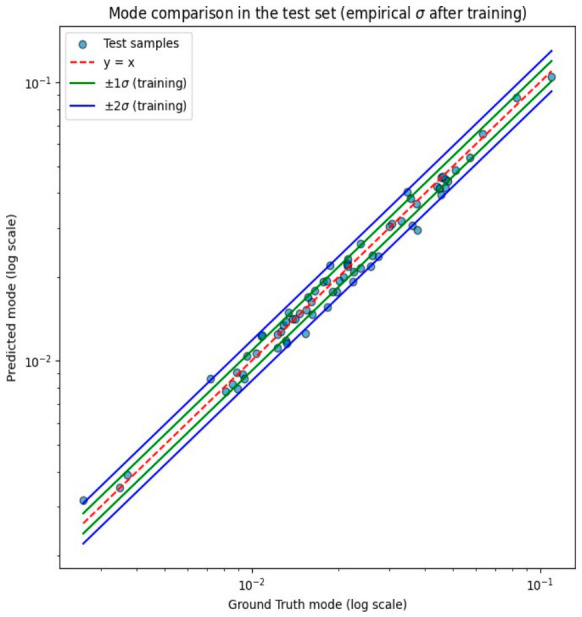
Comparison of model-predicted modes vs. ground truth modes derived from LMS PDFF. Data presented for the test set after the second training stage.

The model’s generalization capability was evaluated on the test set using error intervals derived from the training data (shown in Figure 3). Approximately 70% of test samples fell within ±1σ and 94% within ±2σ, closely matching the expected coverage of a well-calibrated predictive model. Figure 4 compares the predicted PDFF modes with the ground truth modes (LMS PDFF) in the test set. Most predictions fell within the ±2σ uncertainty bands, indicating adequate generalization to unseen data.

In addition, the MAE between the ground truth values and the model predictions on the test set was 0.44 percentage points (pp), with a coefficient of determination of R^2^ = 0.98.

### 3.3. Confidence Intervals for the Test Set

**Figure 5:**
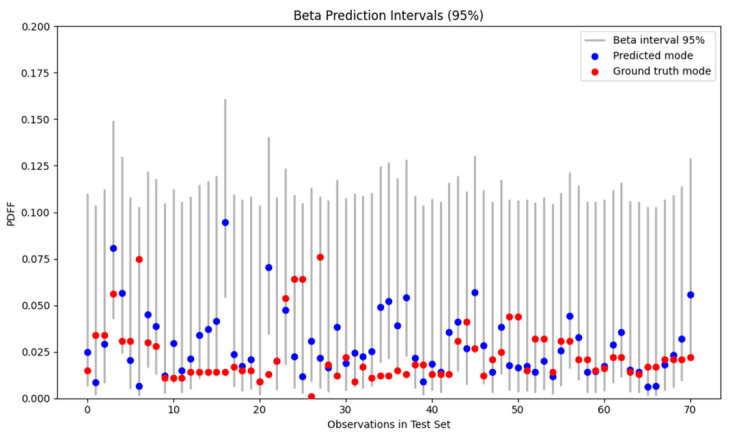
The 95% beta confidence intervals for the test set. Gray vertical lines denote the confidence intervals derived from the model-estimated beta distributions. Blue dots indicate the predicted modes, while red dots represent the ground truth PDFFs.

The confidence intervals derived from the beta distributions are shown in Figure 5. For each observation, the 95% confidence interval calculated based on (*α*″*_i_*, *β*″*_i_*) is displayed together with the predicted-mode PDFF and the corresponding ground truth PDFF. Notably, most ground truth values were within the estimated prediction bounds, indicating good agreement between the model predictions and the observed data.

## 4. Discussion

This study explored the methodological feasibility of using a PNN with a CNN-based architecture to characterize liver fat content from MRI data within a probabilistic framework. The methodology presented allowed the model to achieve a 94% agreement between predicted PDFF values of the test set and ground truth, implying good fat steatosis quantification. The empirical standard deviation obtained was σ=0.0609 on a logarithmic scale for the prediction errors between the predicted values (modes) and the LMS PDFF (ground truth) values. This indicated that the predicted PDFF measurements were in close agreement with ground truth for the test set, with approximately 70% of samples falling within ±1σ and 94% within ±2σ. The resulting confidence intervals reflected the variability in the observed data, providing an uncertainty measure that complemented the estimated PDFF. When expressed in absolute percentage points, the MAE on the test set corresponded to 0.44 pp, with a coefficient of determination of R^2^ = 0.98, both further supporting the quantitative accuracy of the proposed framework.

Direct comparison of the estimated quantitative fat with the existing literature using the proposed PNN and beta distribution framework is difficult. The difficulty arises from previous reports having employed different methodologies, including deterministic regressions, categorical classification with PNNs, segmentation-based pipelines with U-Net-derived fat fraction maps from delineated ROIs [23], and deep learning frameworks for estimating or classifying hepatic fat content from CT data [22]. In contrast, the proposed methodology models liver fat content as a full predictive distribution rather than a single deterministic output, which inherently limits direct metric-based comparisons with deterministic approaches. Nevertheless, a partial numerical reference can be established with Nasir et al. [22], who employed a deep learning framework to predict the PDFF from CT images in a cohort of 94 adults (mean age of 36.11 years, mean PDFF of 3.87%, range of 0–22.3%). When expressed in absolute percentage points, the MAE of 0.44 pp obtained in the present study is lower than the best-case result reported in that work (0.56–3.17 pp). However, this comparison should be interpreted with caution, as the present cohort, even if smaller in size, is prepubertal, with a mean PDFF of 2.69% across the full dataset. Furthermore, the test set exhibited an even lower PDFF mean of ~1.06%; both mean cohort and test set PDFF values are below the 3.87% reported in [22], suggesting that the narrower dynamic range of target values may have contributed to reduced absolute errors. Beyond absolute error metrics, the linear agreement between predicted and ground truth values was further assessed through the coefficient of determination, yielding R^2^ = 0.98 on the test set, which was consistent with the R^2^ = 0.97 reported by Meneses et al. [38] for a multi-decoder water–fat separation network (MDWF-Net), trained on chemical shift-encoded MRI (CSE-MRI) data. Thus, despite differences in imaging modality and population, our study demonstrated a comparable level of predictive accuracy. Taken together, along with the observation that most samples fell within the corresponding confidence intervals, this supports the reliability of the proposed probabilistic framework.

Most existing methods for hepatic fat quantification rely on ROI-based measurements [8,15] or deterministic deep learning formulations [22,23,24,25], typically producing point estimates or categorical outputs. In parallel, probabilistic architectures such as PNNs, Monte Carlo Dropout, and Bayesian CNNs [28], as well as more advanced variants including IPNN-BO [26,33], have been explored in medical imaging for tasks such as liver tumor classification and fat content estimation from ultrasound images [28,33]. These approaches have highlighted the value of uncertainty-aware predictions to improve interpretability and reliability [33]. Although the proposed framework model does not represent fully learned predictive uncertainty (e.g., Bayesian or heteroscedastic modeling), it offers a practical and interpretable way to incorporate the measurement uncertainty within the proposed probabilistic framework. This is particularly relevant given that measurements obtained from a limited number of manually placed ROIs, where PDFF values are sampled and averaged, may be sensitive to the pronounced spatial heterogeneity of hepatic fat. Such heterogeneity has been reported in up to 60% of patients with MASLD [17] and can introduce variability due to regional sampling, which at the same time introduces intrinsic variability in sampling. The clinical value of the proposed methodology lies in providing, in addition to PDFF point estimates, a probabilistic characterization of each measurement through an associated confidence interval. This offers explicit quantification of measurement reliability, which is not typically available from standard PDFF estimation techniques, despite PDFF itself being clinically established. This additional information may be clinically relevant in scenarios where measurement uncertainty influences interpretation, such as borderline cases, longitudinal monitoring, or comparisons across scans or time points.

The trained model presented here is subject to limitations inherent to the dataset, including its high specificity to cohorts comprising exclusively male subjects and prepubertal participants aged 7–9 years old of Latin American ethnicity. In addition, the dataset only included 84 subjects, which restricted generalizability to broader clinical practice. External validation with an independent dataset was not performed due to the lack of availability of such data in Latin America. Consequently, the applicability of the proposed approach beyond the current cohort remains to be determined, particularly given that hepatic steatosis exhibits well-documented variability across age groups, sexes, metabolic profiles, and comorbid conditions [39,40,41]. While the study was conducted on a demographically restricted cohort, the methodology was based on a probabilistic framework that is not inherently population-specific. While certain modeling choices, such as the auxiliary variance and the learned parameters, may vary across datasets and imaging conditions, these differences primarily affect model calibration rather than the underlying approach. However, it should be noted that this variance is partially informed by empirical assumptions derived from the data and may therefore introduce a degree of model-dependent bias in the resulting uncertainty estimates. Thus, the joint estimation of fat fraction and its associated confidence interval remain applicable under comparable conditions, but are not established beyond the current cohort. Generalizability to broader populations, different imaging systems, or varying acquisition settings remains uncertain and warrants further validation across larger or more diverse demographic groups and different clinical contexts, particularly where hepatic fat contents vary substantially. There are additional limitations concerning data acquisition and methodology with respect to generalizability. Data were only acquired on one 3T Skyra Siemens scanner, using a fixed imaging protocol. Furthermore, the proposed methodology requires manual segmentation, which would be rather onerous for large cohorts, and future developments would aim to automate this segmentation step. Finally, ground truth PDFF values were only available without associated uncertainty estimates, which precluded a full probabilistic evaluation of the reference standard.

## 5. Conclusions

This study confirmed the methodological feasibility of using a PNN model with previously segmented liver Dixon MRI data to produce PDFF estimates with confidence intervals. The model learnt to predict the (*α*, *β*) parameters of a beta distribution from single in-phase MRI images, which are widely available across clinical scanners; accordingly, the proposed methodology can be applied to other use cases where similar data are available, provided appropriate retraining is performed. Predictive uncertainty was quantified using empirically estimated variance of the deviation between predicted and reference modes obtained from the training set. The resulting confidence intervals reflected this deviation and provided a complementary measure to the estimated fat steatosis. In contrast to ROI-based approaches, the proposed method relied on whole-image analysis from a single MRI sequence rather than multiple imaging modalities, offering the potential for more comprehensive and clinically informative outputs. Furthermore, modeling liver fat as a beta distribution enabled the estimation of reliable confidence intervals for bounded proportions, which may support more robust clinical decision making in the future if the methodology is verified for other types of populations and scanning protocols.

## Data Availability

The data and analytic methods used in this study remain the property of the individual study sponsors. All deidentified participant data are not openly available presently to allow for privileged use of the data to the funded researchers and may be made available to other researchers upon request to P-WS.

## Supplementary Materials

The following supporting information can be downloaded at: https://www.mdpi.com/article/10.3390/diagnostics16101489/s1.

## References

[1] Younossi Z.M., Koenig A.B., Abdelatif D., Fazel Y., Henry L., Wymer M. (2016). Global epidemiology of nonalcoholic fatty liver disease-Meta-analytic assessment of prevalence, incidence, and outcomes. Hepatology.

[2] Cotter T.G., Rinella M. (2020). Nonalcoholic Fatty Liver Disease 2020: The State of the Disease. Gastroenterology.

[3] Anstee Q.M., Reeves H.L., Kotsiliti E., Govaere O., Heikenwalder M. (2019). From NASH to HCC: Current concepts and future challenges. Nat. Rev. Gastroenterol. Hepatol..

[4] Younossi Z., Tacke F., Arrese M., Chander Sharma B., Mostafa I., Bugianesi E., Wai-Sun Wong V., Yilmaz Y., George J., Fan J. (2019). Global Perspectives on Nonalcoholic Fatty Liver Disease and Nonalcoholic Steatohepatitis. Hepatology.

[5] Gopal P., Hu X., Robert M., Zhang X. (2025). The evolving role of liver biopsy: Current applications and challenges. Heaptology Commun..

[6] Yu Q., Liu Y., Hu P., Gao F., Huang G. (2022). Performance of Imaging Techniques in Non-invasive Diagnosis of Non-alcoholic Fatty Liver Disease in Children: A Systematic Review and Meta-Analysis. Front. Pediatr..

[7] Jayasekera D., Hartmann P. (2023). Noninvasive biomarkers in pediatric nonalcoholic fatty liver disease. World J. Hepatol..

[8] Hutton C., Gyngell M.L., Milanesi M., Bagur A., Brady M. (2018). Validation of a standardized MRI method for liver fat and T2* quantification. PLoS ONE.

[9] Campo C.A., Hernando D., Schubert T., Bookwalter C.A., Pay A.J.V., Reeder S.B. (2017). Standardized Approach for ROI-Based Measurements of Proton Density Fat Fraction and R2* in the Liver. AJR Am. J. Roentgenol..

[10] Reeder S.B., Cruite I., Hamilton G., Sirlin C.B. (2011). Quantitative Assessment of Liver Fat with Magnetic Resonance Imaging and Spectroscopy. J. Magn. Reson. Imaging.

[11] Shumbayawonda E., Beyer C., de Celis Alonso B., Hidalgo-Tobon S., Lopez-Martinez B., Klunder-Klunder M., Miranda-Lora A.L., Thomas E.L., Bell J.D., Breen D.J. (2024). Reference Range of Quantitative MRI Metrics Corrected T1 and Liver Fat Content in Children and Young Adults: Pooled Participant Analysis. Children.

[12] de Celis Alonso B., Shumbayawonda E., Beyer C., Hidalgo-Tobon S., Lopez-Martinez B., Dies-Suarez P., Klunder-Klunder M., Miranda-Lora A.L., Perez E.B., Thomaides-Brears H. (2024). Liver magnetic resonance imaging, non-alcoholic fatty liver disease and metabolic syndrome risk in pre-pubertal Mexican boys. Sci. Rep..

[13] Wang K., Mamidipalli A., Retson T., Bahrami N., Hasenstab K., Blansit K., Bass E., Delgado T., Cunha G., Middleton M.S. (2019). Automated CT and MRI Liver Segmentation and Biometry Using a Generalized Convolutional Neural Network. Radiol. Artif. Intell..

[14] Jeon S.K., Lee J.M., Joo I., Yoon J.H., Lee G. (2023). Two-dimensional Convolutional Neural Network Using Quantitative US for Noninvasive Assessment of Hepatic Steatosis in NAFLD. Radiology.

[15] Elfaal M., Supersad A., Ferguson C., Locas S., Manolea F., Wilson M.P., Sam M., Tu W., Low G. (2023). Two-point Dixon and six-point Dixon magnetic resonance techniques in the detection, quantification and grading of hepatic steatosis. World J. Radiol..

[16] Perspectum (2024). LiverMultiScan v5.1 Guide to Interpreting Liver Tissue Characterization for Physicians.

[17] Starekova J., Hernando D., Pickhardt P.J., Reeder S.B. (2021). Quantification of Liver Fat Content with CT and MRI: State of the Art. Radiology.

[18] Reeder S.B., McKenzie C.A., Pineda A.R., Yu H., Shimakawa A., Brau A.C., Hargreaves B.A., Gold G.E., Brittain J.H. (2007). Water-fat separation with IDEAL gradient-echo imaging. J. Magn. Reson. Imaging.

[19] Lo B.W.Y., Fukuda H. (2024). Bayesian Neural Networks in Predictive Neurosurgery. Computational Neurosurgery.

[20] Vu K.N., Gilbert G., Chalut M., Chagnon M., Chartrand G., Tang A. (2016). MRI-determined liver proton density fat fraction, with MRS validation: Comparison of regions of interest sampling methods in patients with type 2 diabetes. J. Magn. Reson. Imaging.

[21] Pickhardt P.J., Jee Y., O’Connor S.D., del Rio A.M. (2012). Visceral adiposity and hepatic steatosis at abdominal CT: Association with the metabolic syndrome. Am. J. Roentgenol..

[22] Nasir M., Xu Y., Hasenstab K., Yechoor A., Dodhia R., Weeks W.B., Ferres J.L., Cunha G.M. (2025). Liver MRI proton density fat fraction inference from contrast enhanced CT images using deep learning: A proof-of-concept study. PLoS ONE.

[23] Ding J., Cao P., Chang H.C., Gao Y., Chan S.H.S., Vardhanabhuti V. (2020). Deep learning-based thigh muscle segmentation for reproducible fat fraction quantification using fat-water decomposition MRI. Insights Imaging.

[24] Graffy P.M., Sandfort V., Summers R.M., Pickhardt P.J. (2019). Automated Liver Fat Quantification at Nonenhanced Abdominal CT for Population-based Steatosis Assessment. Radiology.

[25] Kim T., Lee D.H., Park E.K., Choi S. (2021). Deep Learning Techniques for Fatty Liver Using Multi-View Ultrasound Images Scanned by Different Scanners: Development and Validation Study. JMIR Med. Inform..

[26] Sindhuja A., Khetavath S. (2025). Prediction and Classification of Fatty Liver Disease Using Probabilistic Neural Networks. J. Intell. Syst. Internet Things.

[27] Molina Pinos D.J. (2024). Diseño de Distribuciones a Priori de Redes Neuronales Bayesianas Basadas en la Solución de su Versión Determinista, in Departamento de Ingeniería Matemática. Master’s Thesis.

[28] Del Corso G., Pascali M.A., Caudai C., De Rosa L., Salvati A., Mancini M., Ghiadoni L., Bonino F., Brunetto M.R., Colantonio S. (2024). ANN uncertainty estimates in assessing fatty liver content from ultrasound data. Comput. Struct. Biotechnol. J..

[29] Gupta A.K., Nadarajah S. (2004). Handbook of Beta Distribution and Its Applications.

[30] Casella G., Berger R.L., Hall C.A. (2024). Statistical Inference.

[31] Hyndman R.J., Athanasopoulos G. (2021). Forecasting Principles and Practice.

[32] Yadav S. (2021). Bayesian deep learning based convolutional neural network for classification of parkinson’s disease using functional magnetic resonance images. SSRN.

[33] Kolli S., Parvathala B.R., Praveen Krishna A.V. (2024). A novel liver tumor classification using improved probabilistic neural networks with Bayesian optimization. e-Prime-Adv. Electr. Eng. Electron. Energy.

[34] Bargagna F., De Santi L.A., Martini N., Genovesi D., Favilli B., Vergaro G., Emdin M., Giorgetti A., Positano V., Santarelli M.F. (2023). Bayesian Convolutional Neural Networks in Medical Imaging Classification: A Promising Solution for Deep Learning Limits in Data Scarcity Scenarios. J. Digit. Imaging.

[35] Sugiyama M. (2016). Chapter 4—Examples of Continuous Probability Distributions. Introduction to Statistical Machine Learning.

[36] Spiegelhalter D.J., Best N.G., Carlin B.P., Linde A. (2014). The Deviance Information Criterion: 12 Years on. J. R. Stat. Soc. Ser. B Stat. Methodol..

[37] Zheng H., Yang Z., Liu W., Liang J., Li Y. (2015). Improving deep neural networks using softplus units. Proceedings of the International Joint Conference on Neural Networks (IJCNN), Killarney, Ireland.

[38] Meneses J.P., Arrieta C., Della Maggiora G., Besa C., Urbina J., Arrese M., Gana J.C., Galgani J.E., Tejos C., Uribe S. (2023). Liver PDFF estimation using a multi-decoder water-fat separation neural network with a reduced number of echoes. Eur. Radiol..

[39] Català-Senent J.F., Hidalgo M.R., Berenguer M., Parthasarathy G., Malhi H., Malmierca-Merlo P., de la Iglesia-Vayá M., García-García F. (2021). Hepatic steatosis and steatohepatitis: A functional meta-analysis of sex-based differences in transcriptomic studies. Biol. Sex Differ..

[40] Chang M., Shao Z., Wei W., Shen P., Shen G. (2023). Sex-specific prevalence and risk factors of metabolic-associated fatty liver disease among 75,570 individuals in eastern China. Front. Endocrinol..

[41] Abu-Freha N., Cohen B., Weissmann S., Hizkiya R., Abu-Hammad R., Taha G., Gordon M. (2022). Comorbidities and Outcomes among Females with Non-Alcoholic Fatty Liver Disease Compared to Males. Biomedicines.

