# Supplementary material for: Hepatic Fat Quantification Using Beta Distribution and a Probabilistic Neural Network in a Prepubertal Male Cohort
Source: Diagnostics (Basel). 2026 May 14;16(10):1489. doi: 10.3390/diagnostics16101489 (PMC13206022; doi:10.3390/diagnostics16101489)
Supplement: Supplementary file 1 [file diagnostics-16-01489-s001.zip › diagnostics-4241608-supplementary.pdf]

Supplementary Information.

1. Parameters of the Beta Distribution.

Given a Beta distribution with mode m ($\alpha > 1$, $\beta > 1$) and variance v:

$$PDFF = mode = \frac{\alpha - 1}{\alpha + \beta - 2} \quad (1)$$

$$var = \frac{\alpha\beta}{(\alpha+\beta)^2(\alpha+\beta+1)} \quad (2)$$

These are the equations to be solved for a system with known m and v.

Solving for α from equation (1):

$$(\alpha + \beta - 2)m = \alpha - 1$$

$$\alpha m + \beta m - 2m = \alpha - 1$$

$$\alpha m - \alpha = -\beta m + 2m - 1$$

$$\alpha(m - 1) = 2m - \beta m - 1$$

$$\alpha = \frac{2m - \beta m - 1}{(m - 1)} = \frac{m\beta(1 - 2m)}{1 - m} \quad (3)$$

To compute β:

From equation (3) we substitute α into equation (2):

$$v = \frac{(\frac{m\beta + (1-2m)}{1-m})\beta}{(\frac{m\beta + (1-2m)}{1-m} + \beta)^2(\frac{m\beta + (1-2m)}{1-m} + \beta + 1)} \quad (4)$$

Therefore, given a variance v, expression (4) is solved numerically to obtain β, and once β is known, equation (3) is used to compute α.
